# Dorsal urethro-cutaneous fistula caused by an impacted stone at the bulbar urethra: Case report

**DOI:** 10.1016/j.ijscr.2021.01.069

**Published:** 2021-01-20

**Authors:** Shakir Saleem Jabali, Ayad Ahmad Mohammed

**Affiliations:** Department of Surgery, College of Medicine, University of Duhok, Kurdistan Region, Iraq

**Keywords:** Urethral stones, Fistula, Urethro-cutaneous fistula, Fistulectomy, Cystoscopy

## Abstract

**Background:**

Penile urethral stones are very and constitutes less than 1% of the urinary tract stones and commoner in males. They are either primary or secondary. The clinical diagnosis require high index of suspicion.

**Case presentation:**

A 30-year-old male was complaining of penile pain, weak urinary stream and dribbling at the end of micturition for 2 months, later on he developed severe dysuria and a tender nodule over the dorsal penile surface. An attempted urethral catheterization was failed. There was no history of urethral trauma or instrumentation. Examination of genitalia revealed a normal meatus with a fistula at dorsal mid penile shaft and the urine were coming out from that opening with surrounding redness and edema with palpable firm nodule in the penile shaft. A pelvic x-ray revealed a mid-urethral radiopaque shadow, cystoscopy revealed an impacted stone in mid-bulbar urethra, attempts of stone extraction was failed. An open ventral urethral incision was made and the stone was removed, dorsal fistulectomy and repair was performed. Foley's catheter was placed and removed later after 21 days. The patient had uneventful postoperative period and the follow up was done up to 6 months with no postoperative complications.

**Conclusion:**

The clinical diagnosis of penile urethral stones require high index of suspicion. Management options are variable depending on impaction site, the size, and associated urethral pathologies. The fistula tract require excised and repair.

## Highlights

• Penile ureteral stones constitutes less than 1% of the urinary tract stones.
• They are either primary (native) or secondary (migrated).
• Secondary urethral stones are commoner and larger in size.
• The clinical diagnosis require high index of suspicion.

## 1 Introduction

Penile urethral stones are very rare clinical presentation and constitutes less than 1% of the urinary tract stones. Most ureteral stones are located in the posterior urethra and they are small in size, giant stones are very rare. They can be diagnosed in both pediatric and adult patients. It is commoner in males while in females it is exceedingly rare due to shorter urethra [[1], [2], [3]].

Primary or called native urethral stones are very rare and arise de novo in the urethra, they are small in size and multiple and usually occur in patients with urethral strictures, diverticulum, infection, hypospadias, and meatal stenosis and are mostly struvite type (magnesium ammonium phosphate), but may be calcium phosphate or calcium carbonate. Secondary urethral stones are commoner than the primary ones and they usually migrate from the upper urinary tract, they are larger than the primary stones and mostly are made from calcium oxalate [1,4,5].

Patients usually present with dysuria, frequency, acute retention of urine, penile pain, perineal pain, hematuria, dribbling, urinary incontinence, pain during ejaculation, and painful coitus. Rarely patients may present with urethro-cutaneous fistula like the presentation in our case. Acquired fistulas are usually caused by trauma, tumors, impacted stones or foreign bodies in the urethra [6,7].

The clinical diagnosis require high index of suspicion, investigations are required to detect other associated stones in the upper urinary tract and other pathologies. Ultrasound is helpful to detect the site of impaction, number of stones in the lower and upper tract, and abnormalities in other parts of the urinary tract like hydronephrosis, infection, or renal stones. Plain X-rays may detect opacifications, their site and the size when the stones are radio-opaque. CT-scan and magnetic resonance imaging yield additional anatomical information and more details about the upper urinary tract that are essential for the diagnosis and to determine the management plan [7,8].

The management options of urethral stones are variable and depend largely on many factors like the location of stone impaction, the size of the stone, and the presence or absence of any associated urethral pathologies. Retrograde manipulation of the stones back into the urinary bladder may be suitable in some patients with small urethral stones, this is followed by litholapaxy or lithotripsy. Anterior urethral stones may be extracted by means of endoscopic removal or by ventral meatotomy when the stone is impacted in the distal shaft of penis [6].

The work of this report case has been reported in line with the SCARE 2020 criteria [9].

## 2 Patient information

A 30-year-old male presented to emergency hospital complaining of penile pain, weak urinary stream and dribbling at the end of micturition for 2 months duration. The pain was constant pain and aggravated with movement, urination, and sexual intercourse and relieved by rest. The color of the urine was normal during this period.

In the last 2 weeks the patient developed severe dysuria along with development of a tender nodule over the dorsal surface of the penis. The patient visited the emergency department. An attempted urethral catheterization was failed at that time.

There was no history of urethral trauma or urethral instrumentation. The past medical and surgical histories were negative, and the drug history were negative.

The family history for any relevant genetic information and psychosocial history was negative.

### 2.1 Clinical findings

**Fig. 1:**
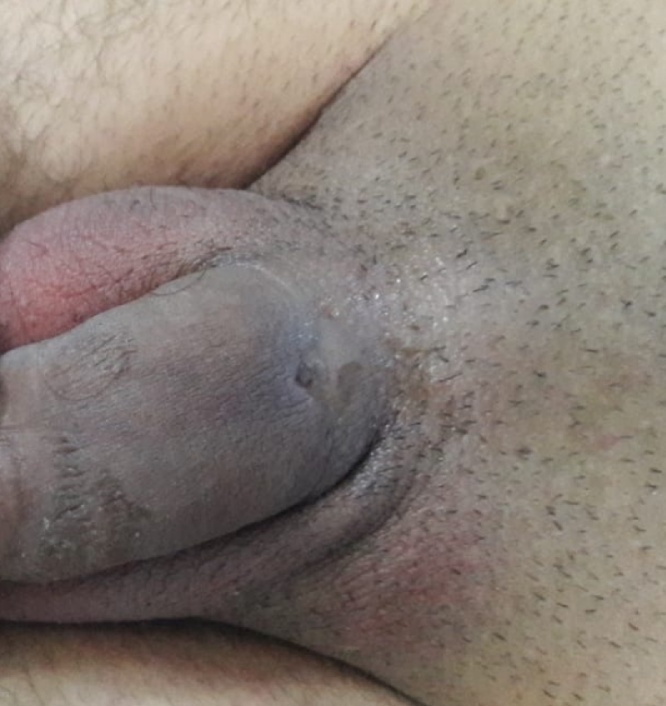
Showing the urethro-cutaneous fistula on the dorsal surface of the penile shaft.

The general examination was unremarkable. Examination of the genitalia revealed a normal urethral meatus but there was a fistula at dorsal mid penile shaft and the urine were coming out from that opening, there was surrounding redness and edema with palpable firm nodule in the penile shaft (Fig. 1).

### 2.2 Diagnostic assessment

**Fig. 2:**
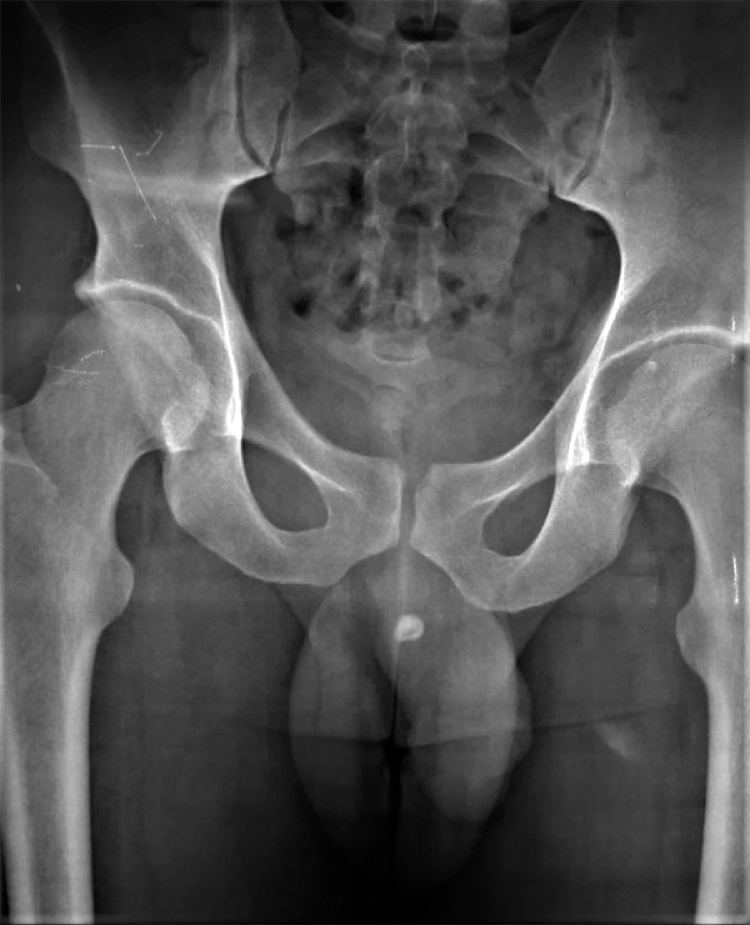
Pelvic X-ray showing the radio-opaque shadow in the penile shaft.

The patient was then sent for investigations, the renal function was normal with normal complete blood count. The urinalysis showed full pus cells and red blood cells in urine. Ultrasonography of abdomen revealed normal bladder wall with normal ureters, no stones and no hydronephrosis. A pelvic x-ray revealed a mid-urethral radiopaque shadow (Fig. 2).

### 2.3 Therapeutic intervention

**Fig. 3:**
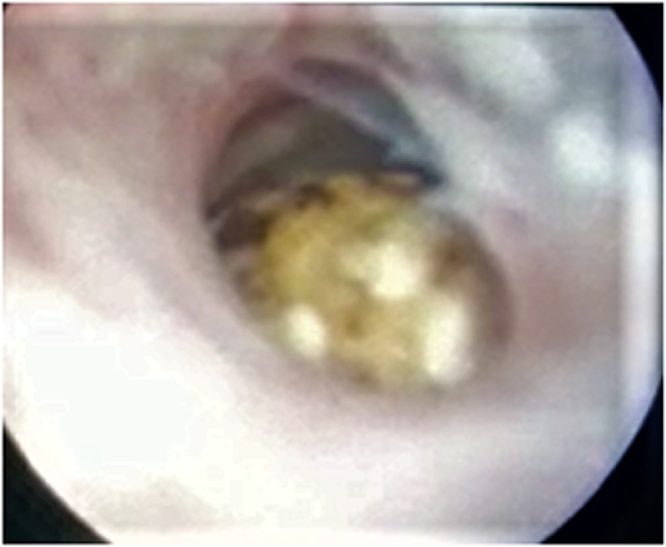
Cystoscopic view of the impacted stone in the mid bulbar urethra.

**Fig. 4:**
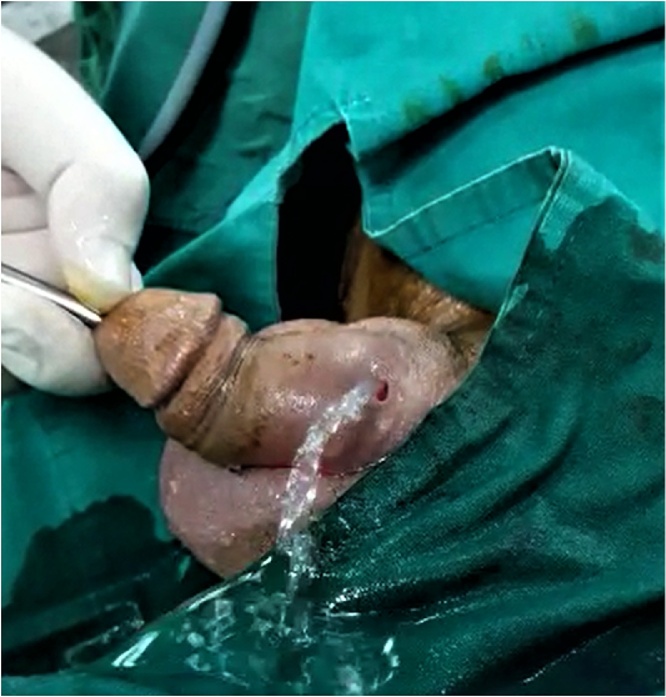
An intraoperative picture showing the spillage of the irrigation fluid mixed with urine from the dorsal penile fistula at the site of stone impaction during cystoscopy.

The patient underwent an emergency cystoscopy which revealed an impacted stone in mid bulbar urethra, attempts of stone extraction was failed, then decision was made to remove the stone by the open procedure (Figs. 3 & 4).

**Fig. 5:**
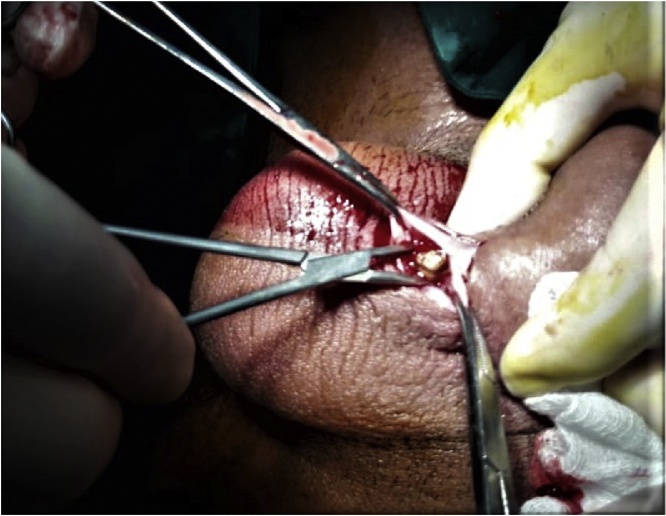
An intraoperative picture showing the ventral incision and the impacted stone in the urethra.

An open urethral incision was made at ventral surface of the penis and then removal of the stone was done along with dorsal fistulectomy and repair was performed. The patients received broad spectrum antibiotics parentally. Foley's catheter was placed and removed later after 21 days (Fig. 5).

### 2.4 Follow-up and outcomes

The patient had uneventful postoperative period and the follow up was done up to 6 months with no postoperative complications. The patients was informed to visit the hospital if he develop any urinary symptoms like difficulty in urination, dysuria, poor stream, and difficult in initiating the urination.

## 3 Discussion

Secondary or migrated urethral stones are commoner than the primary ones. Impacted urethral stones causing urethro-cutaneous fistula is an extremely rare clinical presentation and only few cases are reported in literature [1,5].

Primary stones don’t cause acute symptoms in the majority of patients due to their gradual and slow development, while secondary stones usually cause acute symptoms in most patients. Impacted stones in the urethra may predispose to infection and abscess formation, they may become impacted in the anterior or posterior urethra. The detailed management plan must be addressed before the start of the management [[10], [11], [12]].

It is very important to exclude other associated pathologies that may coexist with urethral stones such as diverticula and strictures. The composition of the stones varies according to the geographical region, in the industrialized societies calcium oxalate stones or cysteine stones are predominant while in the developing countries they are mostly struvite stones or uric acid stones [2,13].

Urethral stones may be easily pushed back to the urinary bladder and then may be managed accordingly either by the endoscopic technique or by open cystolithotomy. Stones which are impacted in the anterior urethra may be pushed outside by gentle antegrade manual milking after generous lubrication of the urethral lumen using xylocaine gel. Sometimes and when the facilities are available stones may be managed using extra-corporeal shock wave lithotripsy, cysto-lithotripsy, electrohydraulic endourethral lithotripsy is also quite effective and less traumatic, and Holmium laser lithotripsy [10,14].

The fistula tract must be removed with debridement of the infected and gangrenous tissues, drainage of associated pus collections, in some patients when the amount of tissue destruction is large, flap reconstruction may be required [6].

Follow up is recommended because some patients may develop stricture at the site of stone impaction especially when the management is delayed and there is associated infection. Strictures are best managed by urethrotomies or urethroplasties [5,15].

## Patient perspective

I had severe pain and had fear when the urine was coming out from the opening in the penis, I was reassured that it is a stone impacted there. After surgery I feel quite better and living my normal life back.

## Declaration of Competing Interest

The authors report no declarations of interest.

## Sources of funding

None.

## Ethical approval

Ethical approval has been exempted by my institution for reporting this case.

## Consent

An informed written consent was taken from the family for reporting the case and the accompanying images.

## Author contribution

Dr Shakir Saleem Jabali and Dr Ayad Ahmad Mohammed contributed to the concept of reporting the case and the patient data recording.

Drafting the work, design, and revision done by Dr Ayad Ahmad Mohammed.

Final approval of the work to be published was done by Dr Ayad Ahmad Mohammed and Dr Shakir Saleem Jabali.

## Registration of research studies

Not applicable.

## Guarantor

Dr Ayad Ahmad Mohammed is guarantor for the work.

## Provenance and peer review

Not commissioned, externally peer-reviewed.
